# Ultra-processed food intake and its association with obesity risk factors, Mediterranean diet, and nutrient intake of adults

**DOI:** 10.3389/fnut.2025.1577431

**Published:** 2025-07-24

**Authors:** Wajd D. Alomari, Noha M. Almoraie

**Affiliations:** Department of Food and Nutrition, Faculty of Human Sciences and Design, King Abdulaziz University, Jeddah, Saudi Arabia

**Keywords:** ultra-processed foods, Mediterranean diet, NOVA classification, obesity, dietary patterns, Saudi adults, nutrient intake

## Abstract

**Purpose:**

Growing awareness highlights ultra-processed food (UPF) as a risk factor for diet-related illnesses. UPF intake is reportedly linked to overweight and obesity statuses; however, this relationship remains unexplored in the Saudi population. In this study, we examined the association between UPF consumption and obesity indicators.

**Methods:**

This study included 190 Saudi participants aged 18–25 years from King Abdulaziz University, Jeddah, Saudi Arabia. Dietary data from two 24-h recalls were classified using the NOVA system. Multiple linear and logistic regression models assessed associations between UPF intake (quartiles) and body mass index (BMI), waist circumference, overweight status (BMI > 25 kg/m^2^) and abdominal obesity (waist circumference ≥88 cm for females; ≥102 cm for males). Models were adjusted for sociodemographic and lifestyle factors.

**Results:**

UPF consumption was associated with a 30.2% higher BMI, a 23% increase in mean waist circumference, and higher odds of having BMI > 25 kg/m^2^ and abdominal obesity (OR = 2.966; 95% CI: 1.86, 4.21; OR = 2.610; 95% CI: 1.46, 3.97, respectively). Increased UPF intake correlated with higher BMI, waist circumference, weight, and hip circumference in both sexes.

**Conclusion:**

Higher UPF consumption is associated with obesity in Saudi adults. Further studies, including intervention trials, are essential to clarify the relationship between UPF intake and health outcomes. Policymakers should promote the consumption of unprocessed or minimally processed foods while limiting UPF intake.

## 1 Introduction

In recent years, there has been a shift in research interests from examining the effects on health status of particular nutrients and food components in the context of overall dietary patterns (1). The Food and Agriculture Organization (FAO) of the United Nations and the World Health Organization (WHO) defined sustainable, healthy diets (2) as “dietary patterns that promote all dimensions of individuals’ health and well-being; have low environmental pressure and impact; are accessible, affordable, safe, and equitable; and are culturally acceptable.” The various components of dietary patterns and their effects on human health and environmental factors have been investigated since the end of the twentieth century (1). The Mediterranean diet (MD) is mainly composed of plant foods, fruit, vegetables, cereals, beans, nuts, and seeds, fresh fruit as the typical daily dessert, olive oil as the principal source of unsaturated fatty acids, and fish. On the other hand, poultry, and alcohol are consumed in low to moderate amounts, and red meat is consumed in low amounts (3). There are four reasons why MD is considered as a sustainable dietary pattern: (1) it a widely recognized for its major health and nutrition benefits, including the prevention of chronic diseases, which decreases public health costs, and overall improvement of well-being; (2) minimal effects on the environment and biodiversity conservation, reduction of pressure on natural resources, and promotion of efforts to mitigate climate change; (3) reduced poverty in agricultural societies, sustainable territorial development, local economic rewards, and decreases in food loss; and (4) increase in and support of high social and cultural food value and identity, and empowerment of consumers (4–6).

Therefore, 91% of studies have referred to the MD as a sustainable dietary pattern (1). Furthermore, the ‘Westernization’ of diets has spread worldwide. This phenomenon reflects a shift from traditional, culturally specific dietary habits toward increased consumption of sugar, sweets, fast food, sugary beverages, red meat, and processed foods (7). Evidence from multiple studies has highlighted the profound influence of Western dietary patterns on shifting nutritional habits of different demographics. The results have demonstrated a link with higher intake of ultra-processed foods (UPFs), highly processed meat, refined grain, and fast food (8). In developed non-Mediterranean countries, such as the United States, the United Kingdom, Canada, and Australia, UPF now account for more than 50% of total dietary energy intake (9–12). Similarly, in other countries of the Mediterranean area, nutritional and lifestyle practices have increasingly shifted from traditional MD to a more “Westernized” food pattern, with negative health consequences (7).

Developing an understanding of these industrially processed foods is of primary importance because the volume and consumption of these foods have risen dramatically everywhere. Recently, food processing has gained attention as a primary contributor to various health problems, including obesity, diabetes, and heart disease, surpassing the nutrient composition of foods or eating patterns (13). A meta-analysis, which investigated the link between UPF consumption and the risk of diabetes, hypertension, dyslipidemia, and obesity based on prospective cohort studies, showed that high UPF intake significantly raised the risk of developing hypertriglyceridemia by 47%, low HDL cholesterol concentration by 43%, diabetes by 37%, hypertension by 32%, and obesity by 32% (14).

Obesity is a significant public health concern, contributing to serious health problems and diminished quality of life while placing a substantial burden on healthcare systems owing to the increased demand for medical care and treatment of obesity-related conditions (15, 16). In 2022, over 2.5 billion adults (43%) were overweight, and more than 890 million (16%) were obese (16). Over the past three decades, the exponential rise in obesity suggests that individual choices and the economic environment influence weight gain (17). Key factors driving this trend include higher income levels, technological advancements in the food industry, increased fast-food consumption, and advertising promoting junk food (18, 19).

Dietary choices play a pivotal role in managing weight. National dietary guidelines emphasize maintaining healthy body weight to prevent obesity (20, 21). Recently, food processing has gained attention as a primary contributor to obesity, surpassing the nutrient composition of foods or eating patterns (18). The NOVA classification system categorizes foods into four groups, identifying UPF as particularly problematic owing to their strong association with obesity risk (22). The definition of UPFs has evolved, emphasizing high levels of sugars, salt, fats, and intentional hyper-palatability for profitability and convenience (22, 23).

A growing awareness of the impact of UPFs on health, along with their association as a risk factor for diet-related diseases, disorders, and conditions, is emerging rapidly. In recent years, technological advancements have significantly transformed the entire food production chain, resulting in greater accessibility and commercialization of UPFs (24). These changes have altered the nutritional content and sensory attributes of foods (18). Although UPFs are nutritionally “empty,” they often contain added substances such as sugars, salt, maltodextrins, protein isolates, artificial sweeteners, high-fructose syrups, and different additives, including colorants, flavorings, and thickeners (18, 22). The UPF ingredients are usually used to enhance the flavor of the products, making the products more palatable, convenient, and economically accessible (17, 23). The link between food processing and the obesity epidemic has gained traction, validated by studies involving over 1 million participants (18). This led to the development of the NOVA food classification system according to the transformation process, which identifies dietary factors associated with obesity risk (18, 25).

The acceptance of the NOVA classification system has expanded, with its principles increasingly incorporated into global dietary recommendations. Several countries now advise limiting UPF intake (26). Numerous scientific societies also support moderating UPF consumption (27, 28). Moreover, proponents and critics of the NOVA classification system acknowledge epidemiological evidence linking higher UPF consumption to increased body mass index (BMI) at the population level. Systematic reviews and meta-analyses (28, 29) have confirmed the association between higher UPF intake and adverse health outcomes. Thus, understanding the implications of UPF consumption is essential for public health initiatives. Recent studies indicate that the obesity epidemic may be fueled by excessive UPF consumption, which is calorie-dense but nutrient-poor (9, 10, 12, 30, 31).

To our knowledge, till date, no study has assessed the relationship between the consumption of UPFs and obesity using the NOVA classification in Saudi Arabia. Therefore, in this study, we aimed to examine the association between UPF intake and obesity indicators among Saudi adults. Furthermore, we also aimed to assess UPF intake in relation to MD adherence and patterns as well as nutrient intake among Saudi adults.

## 2 Materials and methods

### 2.1 Study design

This cross-sectional study was conducted to assess UPF consumption and obesity indicators related to nutrient intake among Saudi adults at King Abdulaziz University, Jeddah, Saudi Arabia. Data were collected between February 2023 and June 2024. Participants were randomly selected from university students across academic years using a complex stratified sampling technique based on college departments. The strata were defined based on the academic unit in which students were enrolled: Foundation Year, Faculty of Education, Faculty of Health Sciences, Faculty of Humanities and Management, and Faculty of Sciences. For each academic unit, official enrollment lists served as the sampling frame. A random number generator was used to select a proportionally representative sample of students from each stratum. Participants were invited via email, which included a survey, and were asked to complete a self-administered questionnaire. The e-mail also included an introduction to the questionnaire that explained the aim of the study and its privacy safeguards (e.g., anonymity). The initial sample included 403 Saudi adults aged 18–25 years that responded to an online survey capturing demographic characteristics, knowledge, and perceptions of UPF. All participants were then invited to voluntarily participate in a follow-up involving two non-consecutive 24-h dietary recalls and self-reported anthropometric measurements. Subsequently, exclusion criteria included the presence of diseases: diabetes, cardiovascular diseases, hypertension, cancer, and eating disorders. No pregnant and breastfeeding women were included. The final analysis included 190 participants (94 males and 96 females).

The Ethics Committee of Human Research of the Faculty of Medicine, King Abdulaziz University, Jeddah, approved the study (Reference No. 25-23). All participants signed in-formed consent forms and were informed of their right to refuse participation or withdraw at any stage without providing a reason.

### 2.2 Obesity indicators

Participants’ weight, height, waist circumference, and hip circumference were self-reported. BMI (weight (kg)/height (m)^2^) and waist circumference (cm) were used as adiposity indices. Overweight was defined as 25.0 kg/m^2^ ≤ BMI < 30.0 kg/m^2^, and obesity was defined as BMI ≥ 30 kg/m^2^ (32). Abdominal obesity was defined as waist circumference of ≥88 cm for females and ≥102 cm for males (33).

Participants self-reported all anthropometric measures on the day after their scheduled Zoom session. This timing ensured alignment with the 24-h dietary recall period. To improve accuracy, detailed measurement instructions were provided to participants immediately before they recorded their measurements. Participants were provided with general instructions to measure their waist and hip circumferences accurately at home. They were advised to take measurements on an empty stomach in the morning. For waist circumference, measurements were performed after several natural breaths at the midpoint between the top of the iliac crest and the lower margin of the last palpable rib in the mid-axillary line, ensuring that the tape was parallel to the floor. Hip circumference was measured at the widest point of the buttocks, keeping the tape level and parallel to the floor. A stretch-resistant tape, wrapped snugly but not constricting, was used to ensure precise measurements. Participants were instructed to remove heavy outer garments, loosen belts, and empty their pockets before measuring. These procedures followed the 2011 WHO guidelines (33).

### 2.3 Dietary assessment

Participants received written instructions to record all foods and beverages they consumed at home and outside during the previous 24 h on two non-consecutive days (one weekday and one weekend). Each page of the instructions provided sections to log the quantity, time, occasion, brand name, and food source of their foods and beverages. Afterwards, recalls were reviewed for exclusions or errors. To minimize errors and biases, the validated USDA Department of Agriculture Automated Multiple-Pass Method was used (34, 35). The two 24-h recalls were coded using the in-house dietary assessment software Diet in Nutrients Out (DINO) (36). Table S1 categorizes all recorded food items according to the NOVA food classification system, which considers the physical, biological, and chemical processes involved in food manufacturing (22).

Each participant was later contacted via Zoom for two meetings (two for each day) to clarify entries and obtain additional details, including brand names, preparation methods, and serving sizes. These interviews followed five sequential passes: (1) asking the respondent to list all foods they consumed that day; (2) prompting memory recall by asking about commonly forgotten items, such as beverages and snacks; (3) collecting more details about the time and occasion of consumption for each food; (4) thoroughly exploring additional information for particular data on amounts, preparation methods, and foods consumed between specified eating occasions; and (5) reviewing all information to identify any missing or forgotten items.

To estimate portion sizes for consumed food items, the validated Photographic Atlas of Food Portions for the Emirate of Abu Dhabi was utilized (37). The food items were classified into four NOVA groups: (1) unprocessed and minimally processed foods (e.g., fresh vegetables and fruits without added salt, sugar, oils, fats, or other substances); (2) processed culinary ingredients (e.g., oils, fats, sugar, salt, and butter); (3) processed foods (e.g., canned fish in oil, fruit in syrup, salted or sugared nuts and seeds); and (4) UPF (e.g., nuggets, ice cream, soft drinks, ready-to-heat pasta dishes). More details about the NOVA classification are available elsewhere (38). The average of the two 24-h recalls for each individual was used to estimate the dietary contribution of UPF as a percentage of total energy intake.

### 2.4 Adherence to MD assessment

From two 24-h recalls of each participant, adherence to the MD was calculated based on the daily consumption of fruits, vegetables, cereals, legumes, fish, meat and meat products, dairy products including cheese, and olive oil. Conformity with the traditional MD was assessed through an MD score (range 0–9 points), as described by Sofi et al., (39). Usually scoring is based on the intake of 9 items: vegetables, legumes, fruit, dairy products, cereals, meat and meat products, fish, alcohol, and olive oil. In this study, only one item (alcohol intake) was not included, because alcoholic beverages are prohibited in Islamic regions.

The eight food groups contributed to a score ranging from 0 points (lowest adherence) to 8 points (highest adherence). For the purposes of this study, participants were categorized into three groups indicating their level of adherence to the MD: low (0–2 points), medium (3–4 points), and high (5–8 points). The MD score is a participant-dependent quality score using dietary intake medians as cut-offs for food components typical of an MD. A value of 0 or 1 is assigned to each component of the score as follows: for components frequently consumed in the traditional MD (vegetables, legumes, fruits, cereals, fish and seafood, as well as olive oil), subjects whose consumption was above the median intake are assigned a value of 1 or are otherwise assigned a score of 0; for components less frequently consumed in the traditional MD (dairy, as well as meat and meat products), subjects whose consumption is equal to or lower than the median are assigned a value of 1 or are otherwise assigned a score of 0.

### 2.5 Covariates

Covariates included sex, age (in years, continuous), marital status (married, divorced/separated/widowed, never married), monthly family income (in Saudi Riyals), living arrangement (with family or alone), smoking or tobacco use (yes or no) and fruit and vegetable intake (yes or no). Sleep duration was categorized as <5, 5–8, and >8 h. Physical activity was estimated using a validated Arabic version of the International Physical Activity Questionnaire-Short Form (IPAQ-SF), which has been previously validated and adopted in Saudi studies (40, 41). In 2000, the questionnaire’s validity and reliability were established through testing across 12 countries (14 sites), with good stability revealed by test–retest reliability (*α* < 0.80) (42). Various studies have shown that IPAQ-SF suits different settings and languages (40, 42). In this study, physical activity levels were classified as low (<600 MET min/week), moderate (600–3,000 MET min/week), and high (>3,000 MET min/week) (43).

### 2.6 Statistical analysis

Statistical analysis was conducted using the Statistical Package for the Social Sciences (SPSS), version 28. Data from the two 24-h recalls were analyzed. Descriptive statistics included frequencies and percentages for categorical variables and means with standard deviations (SD) for continuous variables. The study sample was stratified into quartiles based on the dietary share of UPFs (percentage of total energy intake), with the lowest consumers in the first quartile and the highest in the fourth. Participant characteristics, including demographics, physical activity, smoking status, fruit and vegetable intake, and obesity indicators (BMI and abdominal obesity), were assessed across quartiles of UPF consumption. Differences in these characteristics were evaluated using Pearson’s *χ*^2^ test for categorical variables and unadjusted linear regression models for continuous variables (treating UPF consumption quartiles as an ordinal variable). Linear regression analysis assessed associations between UPF consumption and BMI (kg/m^2^), weight (kg), waist circumference (cm), hip circumference (cm), and nutrient intake. Additionally, linear and logistic regression analyses evaluated the relationship between the dietary contribution of UPFs (quartiles) and nutrient intake and obesity indicators. The percentage of caloric intake from carbohydrates, proteins, and fats, along with the average consumption of fiber (g), sugar (g), sodium (mg), trans fat (g), and saturated fat (g), was evaluated according to UPF consumption quartile using unadjusted linear regression. For obesity indicators, multiple binary logistic regression was used to evaluate associations between relative energy intake from UPF and BMI (as categorized to identify risk of obesity) and waist circumference (as a categorized to identify abdominal obesity). Model 1 included sex, age (continuous), marital status, family income, and living arrangements. Model 2 incorporated additional covariates, such as physical activity level (low, moderate, or high), sleep duration, fruit and vegetable intake, and smoking status (smoker or non-smoker).

The possible relationships between UPF consumption and MD adherence were analyzed by grouping participants according to UPF contribution in the diet and by MD adherence. Thereafter, a general linear model adjusted for age, sex, BMI, living arrangement, physical activity, smoking status, marital status, and daily food intake was conducted to compare MD dietary habits, and UPF types according to the percentage of UPF in the diet, and according to the MD adherence. Because these tests assume normal data distributions, non-distributed data (only food groups such as vegetables and fruits) were transformed into logs; further analyses were performed with the processed data and presented as geometric means with 95% confidence intervals (Cis). *p*-values < 0.05 were considered statistically significant.

## 3 Results

Table 1 presents the characteristics of the study sample, comprising 190 participants. UPFs accounted for 49.2% of the total energy intake among the participants, ranging from 22.1% (0–37.6%) in the lowest quartile of UPF consumption to 73.3% (87.7–100%) in the highest quartile. Participants in the highest quartile of UPF consumption exhibited a significantly higher obesity risk (Q4 = 44.7% vs. Q1 = 14.9%, *p* < 0.001), were more likely to sleep <5 h per day (29.8% vs. 4.3%, *p* < 0.001), and were more often inactive (57.4% vs. 34.0%, *p* = 0.05). Abdominal obesity was also significantly more prevalent among those in the highest quartile (59.6% *vs.* 14.9%, *p* < 0.001). However, no significant differences were observed between demographic characteristics (sex, age, marital status, family income, and residence) and UPF consumption (Table 1). This observation indicates that UPF consumption patterns were consistent across various demographic groups without notable variation. Table 2 examines the relationship between UPF consumption and participants’ anthropometric measurements as obesity indicators. As illustrated, BMI, weight, waist circumference, and hip circumference increased progressively with higher UPF food consumption. Participants in the highest UPF consumption group (Quartile 4; 87.7–100% of energy) had a mean weight of 82.34 ± 26.75 kg, a mean BMI of 28.98 ± 6.66, a mean waist circumference of 95.14 ± 20.34 cm, and a mean hip circumference of 106.53 ± 16.90 cm, compared to those in the lowest consumption group (Quartile 1; 0–37.6% of energy) (*p* < 0.001 for all). These results remained consistent across sexes. Males and females in the highest UPF consumption quartile exhibited significantly greater anthropometric measurements than those in the lowest quartile (*p* ≤ 0.001 for all measurements). Table 3 presents participants’ energy and nutrient intake according to quartiles of relative energy intake from UPFs. Individuals with higher consumption of UPFs reported significantly higher daily caloric intakes, with those in the upper quartile consuming an average of 342.89 kcal/day more than those in the lower quartile (*p* = 0.005). As the relative energy intake from UPFs increased, the consumption of total energy, total fats, trans fats, saturated fats, and sugars also significantly increased (*p* = 0.005 for total energy, *p* < 0.001 for total fat, *p* = 0.018 for trans-fat, *p* = 0.001 for saturated fat, and *p* = 0.030 for sugar). Conversely, higher UPF consumption was associated with a significant decrease in protein intake (*p* = 0.003). However, no statistically significant association was observed between sodium, carbohydrate intake, as well as dietary fiber intake and UPF consumption (*p* > 0.05). Table 4 presents participants’ energy and nutrient intakes according to quartiles of relative energy intake from UPFs by sex. Daily protein intake tended to decrease as UPF consumption increased for both sexes (*p* = 0.016 for males, *p* = 0.018 for females). Additionally, higher relative energy intake from UPFs was associated with significantly increased intake of total fats and saturated fats for both sexes (*p* < 0.05). Among males, increased energy intake from UPFs was also linked to a higher consumption of trans fats and sugars (*p* < 0.05). In contrast, among females, relative energy intake from UPFs was significantly associated with reduced dietary fiber intake (*p* = 0.032). No significant association was found between UPF intake and sodium or carbohydrate intake in both sexes (*p* > 0.05).

**Table 1:** Characteristics of the study sample according to quartile of ultra-processed food consumption (*n* = 190).

Characteristics	Ultra-processed food consumption (% of total daily energy intake)					
	Total (*n* = 190) *n* (%)
Quartile 1 (1) (*n* = 47) *n* (%)	Quartile 2 (*n* = 48) *n* (%)	Quartile 3 (*n* = 48) *n* (%)	Quartile 4 (*n* = 47) *n* (%)	*p*-value (2)
Age (years) (Mean ± SD)	20.98 ± 2.38	20.87 ± 2.30	21.37 ± 2.71	20.71 ± 1.96	20.98 ± 2.48	0.816
Body mass index (BMI) (3)						<0.001
Underweight	21 (11.1)	8 (17.0)	6 (12.5)	4 (8.3)	3 (6.4)	
Normal weight	79 (41.6)	30 (63.8)	23 (47.9)	19 (39.6)	7 (14.9)	
Overweight	55 (28.9)	7 (14.9)	13 (27.1)	14 (29.2)	21 (44.7)	
Obesity	35 (18.4)	2 (4.3)	6 (12.5)	11 (22.9)	16 (34.0)	
Sex						0.454
Male	94 (49.5)	21 (44.7)	21 (43.8)	28 (58.3)	24 (51.1)	
Female	96 (50.5)	26 (55.3)	27 (56.3)	20 (41.7)	23 (48.9)	
Marital status						0.902
Single	184 (96.8)	45 (95.7)	46 (95.8)	47 (97.9)	46 (97.9)	
Married	2 (1.1)	1 (2.1)	0 (0.0)	0 (0.0)	1 (2.1)	
Divorced/widow	4 (2.1)	1 (2.1)	2 (4.2)	1 (2.1)	0 (0.0)	
Family income (Saudi Riyals)						0.857
<5,000	51 (26.8)	14 (29.8)	12 (25.0)	11 (22.9)	14 (29.8)	
5,000–10,000	45 (23.7)	13 (27.7)	11 (22.9)	12 (25.0)	9 (19.1)	
10,000–15,000	29 (15.3)	9 (19.1)	7 (14.6)	5 (10.4)	8 (17.0)	
15,000–20,000	22 (11.6)	5 (10.6)	5 (10.4)	8 (16.7)	4 (8.5)	
>20,000	43 (22.6)	6 (12.8)	13 (27.1)	12 (25.0)	12 (25.5)	
Residence						0.134
Living with family	172 (90.5)	40 (85.1)	44 (91.7)	42 (87.5)	46 (97.9)	
Living alone	18 (9.5)	7 (14.9)	4 (8.3)	6 (12.5)	1 (2.1)	
Sleep duration						0.061
<5 h	35 (18.4)	2 (4.3)	11 (22.9)	8 (16.7)	14 (29.8)	
5–8 h	120 (63.2)	36 (76.6)	30 (62.5)	30 (62.5)	24 (51.1)	
>8 h	35 (18.4)	9 (19.1)	7 (14.6)	10 (20.8)	9 (19.1)	
Smoking						0.225
Yes	38 (20.0)	5 (10.6)	11 (22.9)	13 (27.1)	9 (19.1)	
No	152 (80.0)	42 (89.4)	37 (77.1)	35 (72.9)	38 (80.9)	
Fruits and vegetables intake						0.299
Yes	58 (30.5)	17 (36.2)	18 (37.5)	11 (22.9)	12 (25.5)	
No	132 (69.5)	30 (63.8)	30 (62.5)	37 (77.1)	35 (74.5)	
Physical activity (4)						0.050
Low	83 (43.7)	16 (34.0)	21 (43.8)	19 (39.6)	27 (57.4)	
Moderate	65 (34.2)	14 (29.8)	16 (33.3)	22 (45.8)	13 (27.7)	
High	42 (22.1)	17 (36.2)	11 (22.9)	7 (14.6)	7 (14.9)	
Abdominal obesity (5)						<0.001
Normal	120 (63.2)	40 (85.1)	33 (68.8)	28 (58.3)	19 (40.4)	
Abdominal obesity	70 (36.8)	7 (14.9)	15 (31.3)	20 (41.7)	28 (59.6)	

(1) Mean (range): All, 49.2 (0–100); Q1, 22.1 (0–37.6); Q2, 44.8 (37.6–62.7); Q3, 65.0 (62.7–87.7); Q4, 88.0 (87.7–100). (2) *p*-values for continuous variables are estimated through unadjusted linear regression, treating quartiles as an ordinal variable and Pearson’s *χ*^2^ for categorical variables. (3) Body mass index categorized as underweight (<18.5), normal weight (18.5–24.9), overweight (25–29.9), and obese (≥30) (26). (4) Physical activity was categorized as low (<600 MET min/week), moderate (600–3,000 MET min/week), and high (>3,000 MET min/week). (5) Abdominal obesity is considered as a waist circumference of ≥88 cm for females and ≥102 cm for males (27).

**Table 2:** Association of ultra-processed food consumption and anthropometric measurements of participants.

Variable	Ultra-processed food consumption (% of total daily energy intake)				
	Quartile 1 (1)	Quartile 2	Quartile 3	Quartile 4	*p*-value (2)
	Mean ± SD	Mean ± SD	Mean ± SD	Mean ± SD	
Total (*n* = 190)					
BMI (kg/m^2^)	22.25 ± 4.17	23.45 ± 5.08	25.78 ± 6.77	28.98 ± 6.66	<0.001
Weight (kg)	60.04 ± 14.18	63.71 ± 17.09	72.56 ± 22.69	82.34 ± 26.75	<0.001
Waist Circumferences (cm)	77.36 ± 11.83	80.86 ± 18.73	87.09 ± 21.29	95.14 ± 20.34	<0.001
Hip Circumferences (cm)	92.47 ± 9.84	95.77 ± 17.76	101.20 ± 20.82	106.53 ± 16.90	<0.001
Males (*n* = 94)					
BMI (kg/m^2^)	22.78 ± 4.02	24.76 ± 5.72	26.50 ± 7.70	30.07 ± 6.95	<0.001
Weight (kg)	67.88 ± 13.66	72.69 ± 17.27	79.91 ± 24.19	93.58 ± 30.25	<0.001
Waist Circumferences (cm)	83.95 ± 10.43	87.33 ± 23.022	94.21 ± 24.011	103.68 ± 21.52	<0.001
Hip Circumferences (cm)	91.86 ± 8.49	97.29 ± 22.85	102.93 ± 25.12	109.75 ± 19.54	<0.001
Females (*n* = 96)					
BMI (kg/m^2^)	21.82 ± 4.32	22.44 ± 4.36	24.76 ± 5.23	27.84 ± 6.28	<0.001
Weight (kg)	53.71 ± 11.29	56.72 ± 13.50	62.27 ± 15.82	70.61 ± 16.04	<0.001
Waist Circumferences (cm)	72.03 ± 10.22	75.83 ± 12.90	77.11 ± 11.06	86.22 ± 14.78	<0.001
Hip Circumferences (cm)	92.97 ± 10.96	94.59 ± 12.87	98.77 ± 12.83	103.17 ± 13.22	<0.001

(1) Percentage of energy intake from ultra-processed food, mean (range): All, 49.2 (0–100); Q1, 22.1 (0–37.6); Q2, 44.8 (37.6–62.7); Q3, 65.0 (62.7–87.7); Q4, 88.0 (87.7–100). (2) *p*-values were calculated using the linear regression across ultra-processed food consumption quartiles. BMI, body mass index.

**Table 3:** Consumption of nutrients considering the contribution of each of the quartile consumption of ultra-processed foods.

Nutrients	Ultra-processed food consumption (% of total daily energy intake)				
	Quartile 1 (1)	Quartile 2	Quartile 3	Quartile 4	*p*-value (2)
	Mean ± SD	Mean ± SD	Mean ± SD	Mean ± SD	
Energy(kcal/day)	1774.90 ± 599.32	1809.78 ± 604.25	2005.98 ± 717.41	2117.79 ± 754.92	0.005
Carbohydrate (%)	49.21 ± 8.64	48.85 ± 8.38	50.09 ± 6.76	50.13 ± 5.89	0.412
Protein (%)	18.86 ± 7.41	17.59 ± 5.04	15.85 ± 4.18	15.89 ± 4.12	0.003
Fat (%)	33.35 ± 7.39	36.22 ± 5.59	37.52 ± 5.24	38.49 ± 5.77	<0.001
Trans fat (g)	0.95 ± 0.73	1.16 ± 0.69	1.21 ± 0.84	1.34 ± 0.89	0.018
Saturated fat (g)	21.66 ± 11.38	25.16 ± 9.27	26.07 ± 10.96	29.82 ± 13.39	<0.001
Fiber (g/day)	18.03 ± 8.01	14.71 ± 6.38	15.21 ± 6.20	15.13 ± 6.08	0.065
Sugar (g/day)	69.89 ± 36.22	72.56 ± 25.84	78.95 ± 43.78	84.59 ± 34.18	0.030
Sodium (mg/day)	2944.48 ± 1636.37	2611.42 ± 1491.31	3049.21 ± 2153.26	3183.23 ± 1860.53	0.340

(1) Mean (range): All, 49.2 (0–100); Q1, 22.1 (0–37.6); Q2, 44.8 (37.6–62.7); Q3, 65.0 (62.7–87.7); Q4, 88.0 (87.7–100). (2) *p*-values were calculated using linear regression across ultra-processed food consumption quartiles.

**Table 4:** Consumption of nutrients considering the contribution of each of the quartile consumption of ultra-processed foods (Sex-stratified).

Nutrients	Ultra-processed food consumption (% of total daily energy intake)				
	Quartile 1 (1)	Quartile 2	Quartile 3	Quartile 4	*p*-value (2)
	Mean ± SD	Mean ± SD	Mean ± SD	Mean ± SD	
Males					
Energy (kcal/day)	1,942.65 ± 490.61	2,021.80 ± 572.36	2270.84 ± 730.17	2349.82 ± 660.72	0.014
Carbohydrate (%)	47.66 ± 9.16	49.35 ± 9.47	49.52 ± 5.97	49.93 ± 5.30	0.328
Protein (%)	21.48 ± 9.69	18.09 ± 5.09	16.83 ± 3.80	17.29 ± 3.98	0.016
Fat (%)	33.50 ± 6.82	35.62 ± 5.95	36.28 ± 4.41	37.08 ± 5.78	0.036
Trans fat (g)	1.1070 ± 0.84	1.3109 ± 0.75	1.4670 ± 1.00	1.7246 ± 0.98	0.021
Saturated fat (g)	24.03 ± 12.27	27.07 ± 9.43	28.97 ± 12.96	32.55 ± 12.93	0.017
Fiber (g/day)	17.67 ± 5.83	15.74 ± 6.59	16.36 ± 6.46	16.47 ± 5.85	0.624
Sugar (g/day)	70.64 ± 32.69	76.42 ± 25.00	88.97 ± 49.83	89.98 ± 35.00	0.048
Sodium (mg/day)	2799.67 ± 1270.93	2708.25 ± 1205.25	3417.14 ± 2482.91	3590.95 ± 2304.51	0.094
Females					
Energy (kcal/day)	1639.41 ± 652.39	1644.88 ± 586.07	1635.17 ± 517.09	1875.67 ± 784.36	0.232
Carbohydrate (%)	50.46 ± 8.16	48.47 ± 7.59	50.89 ± 7.82	50.33 ± 6.57	0.783
Protein (%)	16.74 ± 3.92	17.20 ± 5.06	14.48 ± 4.39	14.43 ± 3.81	0.018
Fat (%)	33.22 ± 7.96	36.70 ± 5.37	39.26 ± 5.90	39.95 ± 5.50	<0.001
Trans fat (g)	0.8273 ± 0.62	1.0518 ± 0.64	0.8648 ± 0.32	0.9552 ± 0.57	0.683
Saturated fat (g)	19.75 ± 10.45	23.68 ± 9.04	22.00 ± 5.32	26.98 ± 13.54	0.030
Fiber (g/day)	18.33 ± 9.52	13.92 ± 6.21	13.61 ± 5.58	13.74 ± 6.14	0.032
Sugar (g/day)	69.28 ± 39.48	69.55 ± 26.55	64.91 ± 29.28	78.96 ± 33.12	0.407
Sodium (mg/day)	3061.44 ± 1898.13	2536.10 ± 1699.84	2534.11 ± 1493.31	2757.79 ± 1781.10	0.554

(1) Mean (range): All, 49.2 (0–100); Q1, 22.1 (0–37.6); Q2, 44.8 (37.6–62.7); Q3, 65.0 (62.7–87.7); Q4, 88.0 (87.7–100). (2) *p*-values were calculated using linear regression across ultra-processed food consumption quartiles.

An increase in the consumption of UPFs was significantly associated with all outcomes in crude and multivariable models adjusted for potential confounders (Table 5). Table 5 presents the results of multiple logistic regression analysis for the association between UPF intake and obesity risk as assessed by BMI and waist circumference. Compared with participants in the lowest quartile of relative energy intake from UPFs, the odd ratios for BMI and waist circumference were increased for participants in the highest quartile (quartile 4; 87.7–100% of energy). In multivariable analyses of Models 1 and 2, higher UPF consumption was associated with higher odds of having a BMI of ≥25 kg/m^2^ and abdominal obesity (OR = 2.966; 95% CI: 1.86, 4.21; OR = 2.610; 95% CI: 1.46, 3.97, respectively). A significant association was observed between increased UPF consumption and each outcome (*p* < 0.001), as presented in Table 5.

**Table 5:** Crude and adjusted analyses between energy contribution (%) of the consumption of ultra-processed food and obesity indicators.

Variable	Ultra-processed food consumption (% of total energy)				
	Quartile 1 (1)	Quartile 2	Quartile 3	Quartile 4	*p*-value trend (4)
	OR (95%CI)	OR (95%CI)	OR (95%CI)	OR (95%CI)	
BMI ≥ 25 kg/m^2^					
Crude	1.0 (ref)	1.321* (0.32, 2.43)	1.838* (0.86, 2.94)	2.992* (1.97, 4.15)	<0.001
Model 1 (2)	1.0 (ref)	1.323* (0.28, 2.47)	1.843* (0.83, 2.98)	2.956* (1.89, 4.15)	<0.001
Model 2 (3)	1.0 (ref)	1.316* (0.25, 2.48)	1.914* (0.86, 3.08)	2.966* (1.86, 4.21)	<0.001
Abdominal obesity (5)					
Crude	1.0 (ref)	1.276* (0.12, 2.61)	1.864* (0.76, 3.18)	2.763* (1.67, 4.08)	<0.001
Model 1	1.0 (ref)	1.228 (0.049, 2.594)	1.840* (0.71, 3.17)	2.652* (1.44, 3.85)	<0.001
Model 2	1.0 (ref)	1.204 (0.01, 2.58)	1.842* (0.69, 3.19)	2.610* (1.46, 3.97)	<0.001

(1) Mean (range): All, 49.2 (0–100); Q1, 22.1 (0–37.6); Q2, 44.8 (37.6–62.7); Q3, 65.0 (62.7–87.7); Q4, 88.0 (87.7–100). (2) Model 1 adjusted for demographic information (age, sex, marital status, living arrangements, and income level). (3) Model 2 adjusted for Model 1 + physical activity + smoking + sleep duration + fruit and vegetable consumption. (4) *p*-values were calculated using logistic regression of the total percentage of ultra-processed food consumption, with all models showing *p* < 0.001. (5) Abdominal obesity is considered as a waist circumference ≥88 cm for females and ≥102 cm for males (27). BMI, body mass index. *p-value < 0.05.

**Figure 1:**
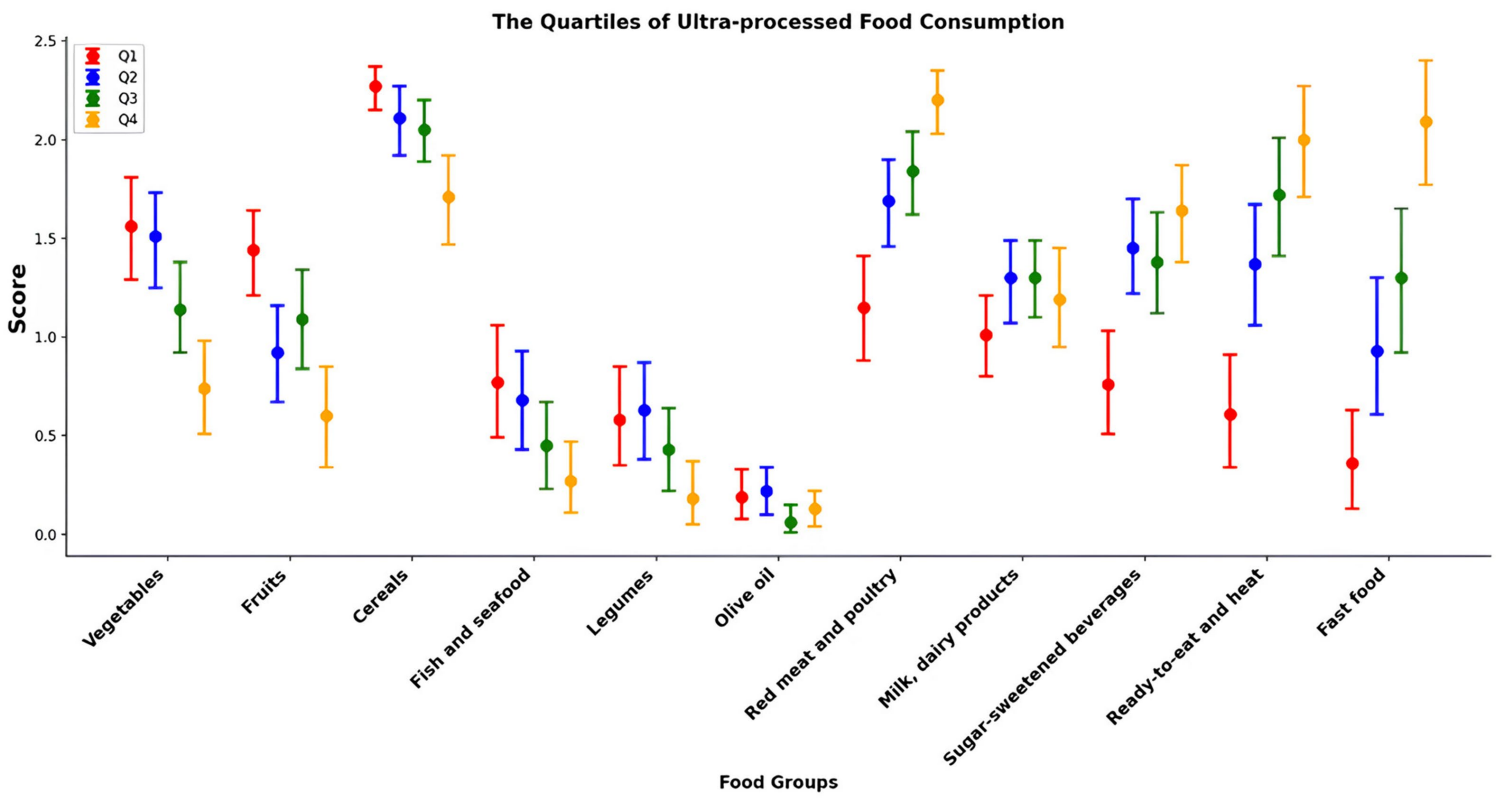
Contributors to Mediterranean diet and ultra-processed foods: food groups by ultra-processed food quartiles.

**Figure 2:**
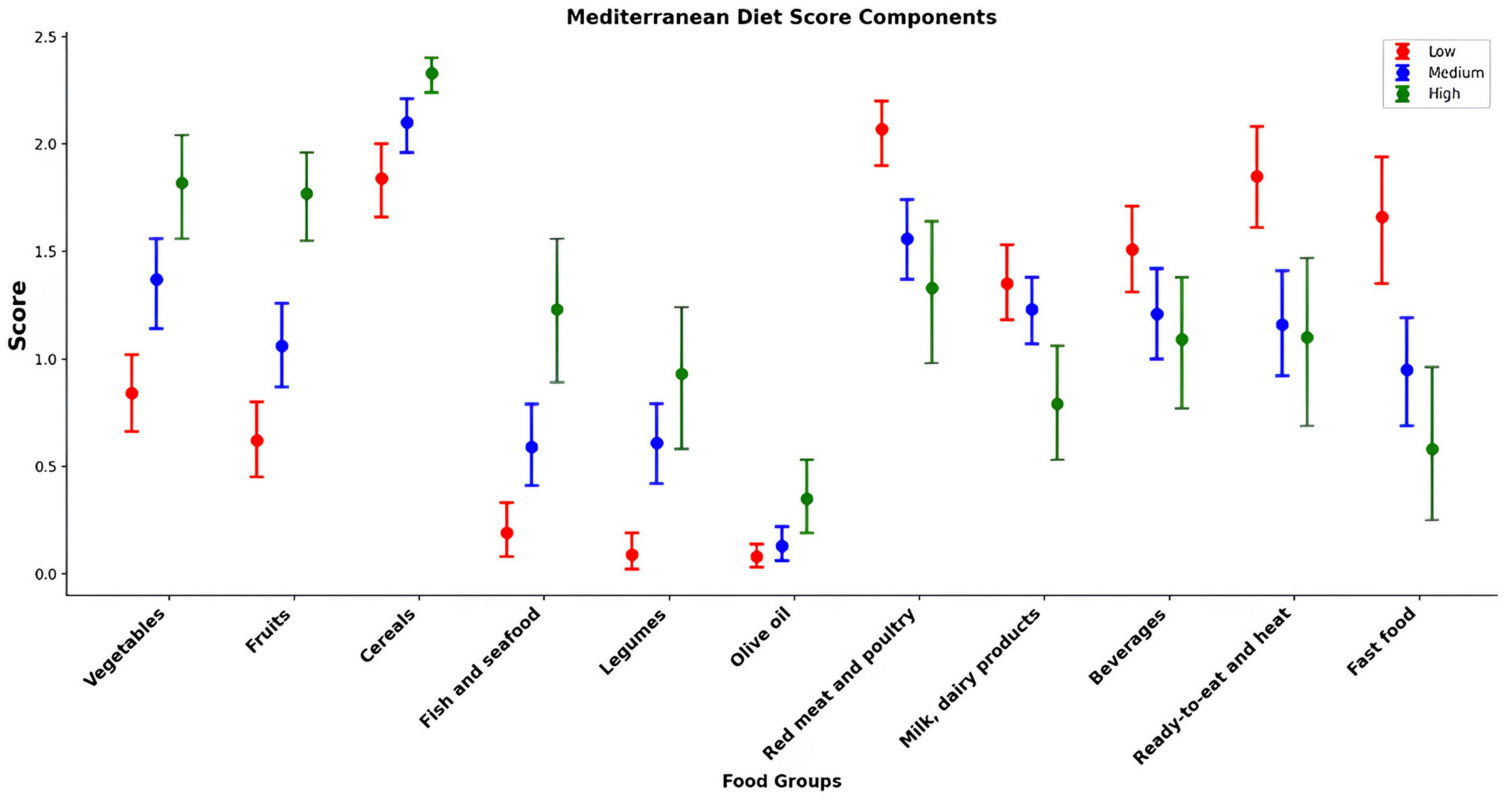
Contributors to Mediterranean diet and ultra-processed foods: food groups by Mediterranean diet adherence score.

Food consumption (g/day) according to the percentage of total daily energy intake of UPF in the diet is reported in Table 6 and Figure 1. After adjustment for possible confounding factors such as age, sex, daily food intake, BMI, marital status, physical activity, and smoking, participants in the highest UPF quartile (quartile 4; 87.7–100% of energy) showed a significantly lower consumption of fruits, vegetables, cereals, legumes, and fish and seafood than those in the first quartile (Quartile 1; 0–37.6% of energy), and higher consumption of red meat and poultry, ready-to-eat and heat, fast food (*p* < 0.001 for all). A similar trend was observed for UPF beverages, with participants in the highest UPF quartile reporting a higher consumption of soft drinks, energy drinks, fruit drinks, fruit juices, instant coffee and coffee drinks than participants in the lowest UPF quartile. No significant differences were observed for milk and dairy products, as well as olive oil consumption. Meanwhile, Table 7 and Figure 2 examine the relationship between adherence levels to the MD and the consumption of MD and UPF food consumption (g/day) among participants. As illustrated, the intake of MD types, fruits, vegetables, cereals, legumes, olive oil, and fish and seafood increased progressively with higher adherence to MD. Conversely, the consumption of dairy products and red meat decreased with higher adherence to the MD, which provides a significant positive scoring when reporting the consumption of food groups in line with the MD and significant negative scoring related to the consumption of food groups, not in line with the MD (i.e., meat and dairy products). A similar trend was observed for the UPF diet: the higher the participants’ adherence to MD, the lower their consumption of UPFs, fast food, ready-to-eat meals, and UPF beverages.

**Table 6:** Consumption of the Mediterranean diet score dietary components and the main types of ultra-processed foods, considering the contributions from each quartile of ultra-processed food consumption.

Variable	Ultra-processed food consumption (% of total daily energy intake)				
	Quartile 1 (1)	Quartile 2	Quartile 3	Quartile 4	*p*-value (2)
Vegetables	1.56 (1.29, 1.81)	1.51 (1.25, 1.73)	1.14 (0.92, 1.38)	0.74 (0.51, 0.98)	<0.001
Fruits	1.44 (1.21, 1.64)	0.92 (0.67, 1.16)	1.09 (0.84, 1.34)	0.60 (0.34, 0.85)	<0.001
Cereals (Grains and pasta)	2.27 (2.15, 2.37)	2.11 (1.92, 2.27)	2.05 (1.89, 2.20)	1.71 (1.47, 1.92)	<0.001
Fish and seafood	0.77 (0.49, 1.06)	0.68 (0.43, 0.93)	0.45 (0.23, 0.67)	0.27 (0.11, 0.47)	0.002
Legumes	0.58 (0.35, 0.85)	0.63 (0.38, 0.87)	0.43 (0.22, 0.64)	0.18 (0.05, 0.37)	0.006
Olive oil	0.19 (0.08, 0.33)	0.22 (0.10, 0.34)	0.06 (0.01, 0.15)	0.13 (0.04, 0.22)	0.228
Red meat and poultry	1.15 (0.88, 1.41)	1.69 (1.46, 1.90)	1.84 (1.62, 2.04)	2.20 (2.03, 2.35)	<0.001
Milk, dairy products	1.01 (0.80, 1.21)	1.30 (1.07, 1.49)	1.30 (1.10, 1.49)	1.19 (0.95, 1.45)	0.169
Beverages (3)	0.76 (0.51, 1.03)	1.45 (1.22, 1.70)	1.38 (1.12, 1.63)	1.64 (1.38, 1.87)	<0.001
Ready-to-eat and heat (4)	0.61 (0.34, 0.91)	1.37 (1.06, 1.67)	1.72 (1.41, 2.01)	2.00 (1.71, 2.27)	<0.001
Fast food (Pizza and burgers)	0.36 (0.13, 0.63)	0.93 (0.61, 1.30)	1.30 (0.92, 1.65)	2.09 (1.77, 2.40)	<0.001

Data are reported as geometric mean and 95%confidence interval (CI). (1) Mean (range): All, 49.2 (0–100); Q1, 22.1 (0–37.6); Q2, 44.8 (37.6–62.7); Q3, 65.0 (62.7–87.7); Q4, 88.0 (87.7–100). (2) *p*-values were calculated using linear regression adjusted for age, sex, BMI, marital status, physical activity, smoking, and total energy intake. (3) Soft drinks, energy drinks, fruit drinks, fruit juices, instant coffee and coffee drinks. (4) Packaged pre-prepared meals, canned soups and beans, French fries (frozen), and noodles.

**Table 7:** Consumption of the Mediterranean diet score dietary components and the main types of ultra-processed foods, considering the contribution of each level of adherence to the Mediterranean diet.

Variable	Level of adherence to the Mediterranean diet (MD)			
	Low (1) (*n* = 76)	Moderate (*n* = 81)	High (*n* = 33)	*p*-value (2)
Vegetables	0.84 (0.66, 1.02)	1.37 (1.14, 1.56)	1.82 (1.56, 2.04)	<0.001
Fruits	0.62 (0.45, 0.80)	1.06 (0.87, 1.26)	1.77 (1.55, 1.96)	<0.001
Cereals (Grains and pasta)	1.84 (1.66, 2.00)	2.10 (1.96, 2.21)	2.33 (2.24, 2.40)	<0.001
Fish and seafood	0.19 (0.08, 0.33)	0.59 (0.41,0.788)	1.23 (0.89, 1.56)	<0.001
Legumes	0.09 (0.02, 0.19)	0.61 (0.42, 0.79)	0.93 (0.58, 1.24)	<0.001
Olive oil	0.08 (0.03, 0.14)	0.13 (0.06, 0.22)	0.35 (0.19, 0.53)	0.003
Red meat and poultry	2.07 (1.90, 2.20)	1.56 (1.37, 1.74)	1.33 (0.98, 1.64)	<0.001
Milk, dairy products	1.35 (1.18, 1.53)	1.23 (1.07, 1.38)	0.79 (0.53, 1.06)	<0.001
Beverages (3)	1.51 (1.31, 1.71)	1.21 (1.00, 1.42)	1.09 (0.77, 1.38)	0.026
Ready-to-eat and heat (4)	1.85 (1.61, 2.08)	1.16 (0.92, 1.41)	1.10 (0.69, 1.47)	<0.001
Fast food (Pizza and burgers)	1.66 (1.35, 1.94)	0.95 (0.69, 1.19)	0.58 (0.25, 0.96)	<0.001

Data are reported as geometric mean and 95%confidence interval (CI). (1) Mean (range): Low = 0–2; Medium = 3–4; High = 5–8. (2) *p*-values were calculated using linear regression adjusted for age, sex, BMI, marital status, physical activity, smoking, and total energy intake. (3) Soft drinks, energy drinks, fruit drinks, fruit juices, instant coffee and coffee drinks. (4) Packaged pre-prepared meals, canned soups and beans, French fries (frozen), and noodles.

## 4 Discussion

In this study, higher UPF consumption was associated with greater BMI, waist circumference, and increased odds of obesity and abdominal obesity. Higher UPF consumption was associated with excess weight and waist circumference in both sexes. Additionally, higher UPF consumption was associated with lower adherence to MD, and the intake of MD food types (fruits, vegetables, cereals, legumes, olive oil, and fish and seafood) increased progressively with higher adherence to MD and lower intake of UPF.

Several similar cross-sectional studies have reported comparable findings. Individuals with higher UPF consumption had significantly greater odds of being overweight or obese to varying degrees (12, 30, 44). Prospective cohort studies have also identified a causal relationship between higher UPF consumption and overweight or obesity (10, 45, 46). Machado et al. (12) revealed a significant link between UPF consumption and obesity indicators in a cross-sectional analysis of 7,411 Australians aged ≥20 years, as well as a higher prevalence of obesity with a BMI of 0.97 kg/m^2^ and waist circumference of 1.92 cm in those with the highest UPF consumption. Similarly, Rauber and colleagues conducted a prospective cohort study in a United Kingdom adult population (aged ≥18 years), finding that a 10% increase in UPF consumption was linked to a 0.38 kg/m^2^ increase in BMI, a 0.87 cm increase in waist circumference, and an 18% higher likelihood of obesity, even after adjusting for sociodemographic and lifestyle factors (12). This study also confirmed that individuals in the highest UPF consumption quartile (Quartile 4; 87.7–100% of total energy intake) had a 30.2% increase in mean BMI and a 23% increase in mean waist circumference. Furthermore, with each incremental rise in UPF intake, the odds of overweight status and abdominal obesity increased. A recent prospective cohort study of 10,260 adults from the NutriNet-Santé cohort (2009–2019) in France reported similar results (46). After adjusting for age, sex, educational level, lifestyle factors, and energy intake, the study observed a positive association between UPF consumption and BMI increases. Specifically, a 10% increase in UPF intake was associated with a higher risk of overweight (HR = 1.11, 95% CI: 1.08, 1.14) and obesity (HR = 1.09, 95% CI: 1.05, 1.13), both statistically significant (*p* < 0.001) (46). This study found no significant associations between UPF consumption and sociodemographic factors, including sex, age, marital status, family income, or residence. Similarly, a recent systematic review analyzing 1,131 results from various observational and nationally representative studies reported that household status and sex were generally unrelated to UPF intake. However, the review noted that higher UPF consumption was more prevalent among younger individuals, urban residents, and those who were unmarried, single, separated, or divorced (14). Supporting these findings, another study observed stable UPF consumption patterns over time. From 2008 to 2019, there were no significant linear trends in UPF intake across sociodemographic groups within the United Kingdom population. This analysis, based on dietary data collected through a four-day food record from the National Diet and Nutrition Survey (NDNS), assessed the dietary share of foods categorized using the NOVA classification (47). These findings suggest that UPF consumption is influenced less by individual demographic characteristics and more by broader lifestyle and environmental factors.

Several factors explain the link between increased UPF intake, energy overconsumption, and obesity. Firstly, UPF is more energy-dense compared to traditional diets (18, 48). Additionally, UPF contains higher levels of saturated fat, added sugar, and salt while being lower in fiber, minerals, and vitamins (47, 48). This nutritional composition results from various industrial processes, such as water removal, which extends shelf life, reduces transportation costs, and increases energy density per serving (45). Nutritionally, UPF also contains greater amounts of trans and saturated fats and synthetic additives. This study found a notable positive association between relative energy intake from UPF and total energy consumption, as well as total, saturated, and trans fats intake, along with sugars, accompanied by a decrease in protein intake. Similar findings have been reported in studies conducted in different countries. Numerous nationwide studies consistently demonstrate a substantial association between UPF consumption and the nutritional insufficiency of diets (9, 25, 30, 45, 49). A study conducted in Chile with 5,753 individuals found that as UPF intake increased, there was a notable decline in protein, fiber, and essential micronutrients, alongside a rise in carbohydrates, added sugars, and saturated fats (49). Similar findings were reported in a 2018 study by Juul et al. involving 15,977 adults in the United States. This study confirmed that higher UPF consumption was associated with increased carbohydrates, total fat, and total sugar. Conversely, a significant negative association was observed between fiber and protein content (9). In a prospective Spanish cohort, the SUN (University of Navarra Follow-Up) study reported similar results (37). The increased UPF intake was linked to higher total fat, lower protein and total fiber, and reduced adherence to the Mediterranean diet. The authors suggested that UPF consumption may raise the risk of being overweight and obesity by increasing overall calorie intake, added and free sugars, and fats while providing an inappropriate nutrient ratio, contributing to fat accumulation (45).

However, in this study, no positive association was observed between dietary intake of sodium and carbohydrates and UPF consumption. Similar findings were reported in previous studies in the United States (25), Korea (30), and Portugal (50), which noted no association between increased UPF intake and sodium consumption. Additionally, comparable results were observed in Canada (51) and Brazil (52) regarding carbohydrates. This variation may stem from differences in the primary sources of sodium and carbohydrates across regions (25, 30, 50–52). In Saudi Arabia, traditional dishes predominantly rely on rice and whole-grain wheat as staples (53). According to Al-Mssallem (53), popular wheat-based dishes such as Harees (whole grain wheat cooked with meat), Mataziz, Qorsan, Marqooq (whole wheat dough with vegetables and meat), Gerish (cracked wheat cooked with vegetables and meat), Marassia, Aseedah, Maamool Tamer, Qors Tamer, and Klaija are rich in carbohydrates and may influence sodium content. Notably, this study did not classify most of these traditional foods as UPFs.

Research has reported potential differences between the sexes regarding the link between UPF consumption and obesity (9, 30, 44). In this study, we found that males and females with the highest UPF consumption had significantly greater mean waist circumferences, BMI, and body weight. A dose–response relationship was observed in both sexes, with increased UPF consumption being associated with a 32% increase in BMI in males and a 27.6% increase in females. Similar findings were reported in a previous study in 2021 (10), which confirmed that UPF consumption was associated with increased BMI, waist circumference, and obesity prevalence in both sexes. The study also revealed a dose–response relationship, where a 10% rise in UPF consumption corresponded to an 18% higher obesity prevalence in males and a 17% increase in females. Furthermore, as the relative energy intake from UPF increased, daily protein intake decreased, while the intake of total fats and saturated fats significantly increased in both sexes.

The results of this study indicate that for both sexes, dietary fat intake increases with higher UPF consumption, which may be related to higher calorie intake and weight gain. UPF may promote weight gain through their nutritional intake by displacing low-energy, nutrient-dense, unprocessed, and minimally processed foods from the diet and encouraging poor dietary habits (48, 51, 52). UPF accessibility, price, convenience, and aggressive marketing encourage involuntary overeating and continuous snacking, potentially replacing less processed, more nutrient-dense foods in the diet (22, 51). Additionally, the high-intensity flavoring in UPF may promote overeating and override endogenous satiety responses (14).

In addition, UPFs are, on average, more energy-dense than unprocessed and minimally processed foods and culinary preparations based on minimally processed foods (17, 31). As human satiety mechanisms are more sensitive to volume than energy content, foods with higher energy density may facilitate excessive energy intake (54, 55). A recent study among 224 Brazilian adults examined the association between UPF consumption and normal weight obesity (NWO). Participants were divided into two groups: the NWO group (159 individuals) with a high body fat percentage (%BF) and the non-NWO group (65 individuals) with normal %BF (56). The study showed a significant relationship between the type of food consumed and overall dietary quality. Individuals in the NWO group had a lower total energy intake from fresh or minimally processed foods, such as rice, beans, and fruits, compared to the non-NWO group. Additionally, the NWO group consumed more processed meats. In contrast, the non-NWO group reported higher dietary fiber intake and greater consumption of essential nutrients, including protein, carbohydrates, fiber, calcium, iron, sodium, and sugar, all derived from non-UPF. These findings indicate that diets rich in minimally processed foods are associated with improved nutrient quality, underscoring the importance of unprocessed foods in fostering healthier dietary patterns (56).

Currently, a higher adherence to MD is known to reduce the risk of all-cause mortality, cardiovascular diseases, coronary heart disease, myocardial infarction, overall cancer incidence, neurodegenerative diseases, and diabetes (57). In line with the literature (56, 58–60), the results obtained from this study confirm a significant inverse association between UPF consumption and adherence to MD, highlighting that higher UPF consumption is associated with lower adherence to MD and MD food.

This could be explained as a nutritional transition from fresh meals and dishes that are part of a traditional cuisine toward a higher intake of ready-to-consume and hyper-palatable food and beverages products. Indeed, an impact on the intake of some of the foods known to part of the MD was reported owing to the high UPF consumption. Specifically, participants with greater UPF intake reported significantly lower consumption of fruits, vegetables, cereals, legumes, and fish and seafood and higher consumption of red meat, ready-to-eat and heat, fast food and UPF beverages, in contrast to participants with a lower UPF consumption. Similar findings were reported by other studies of Mediterranean populations, such as those in Spain (58), France (61), and Italy (62). To better understand the association between UPF intake and MD adherence in our study participants, we also investigated the possible influence of individual UPF consumption on the MD adherence score, observing that the consumption of fast food, processed meats, soft and energy drinks, and ready-to-eat and heat foods negatively influenced the MD adherence score. Interestingly, most of these foods were major contributors to UPF consumption and are present in all countries of the world, supporting the hypothesis of a nutritional transition from a sustainable diet such as MD to Westernized dietary patterns due to increased UPF consumption. There is an urgent need to raise awareness of the negative health effects of excessive UPF consumption and new public-health strategies to prevent the progressive loss of traditional diets.

Furthermore, people are more likely to overlook nutritional considerations when consuming foods, and urbanization has not been accompanied by advances in nutrition understanding (44). This trend can lead to increased UPF consumption and a higher overweight or obesity prevalence. Public health policies have significantly influenced individuals’ food patterns, necessitating multiple approaches to address the global obesity pandemic (17). Limiting UPF consumption is essential as these foods contain excessive levels of additives, sugars, and fats (22, 23, 63). Further research is needed to examine the relationships between urbanization, food consumption, and nutritional health, alongside the development of suitable nutritional recommendations. Our findings underscore the importance of advancing sustainable and healthy food standards, requiring behavioral and structural adjustments. Food systems should prioritize healthier eating habits and enhance the availability of nutritious foods.

This study has several strengths. To our knowledge, this is the first study to examine the association between UPF consumption, obesity, and adherence to MD in Saudi Arabia. Furthermore, this study used the NOVA food classification system, recognized as a valid tool for nutrition and public health research and policy (63). The most updated version of NOVA, which separately classifies processed foods and UPFs, was used.

Some limitations should also be noted. Owing to the cross-sectional nature of the data, temporality and causation cannot be established, and reverse causality cannot be excluded. Additionally, residual confounding could not be fully eliminated, although adjustments were made for potential confounding factors. Furthermore, while standardized instructions and follow-up support were implemented to enhance accuracy, the self-reported nature of anthropometric data remains a limitation owing to potential misreporting. As is common in nutritional epidemiological studies, dietary assessment by 24-h recall is an imprecise measure of diet, and the foods consumed on the assessment day may not fully represent an individual’s regular diet. In this study, dietary intakes were estimated using two non-consecutive 24-h recalls, which may still fall short in representing participants’ typical habitual dietary intake. However, this study used the AMPM method, a validated approach to dietary data collection that has been shown to reduce bias (34, 35). Lastly, the findings cannot be generalized to the entire Saudi population, because the sample was drawn exclusively from Jeddah and comprised entirely of young adults aged 18–25 years with an educational background. Although this offers useful insights, the results might not be typical of the broader Saudi adult population, which also includes individuals with non-academic or professional backgrounds, those from older age groups, and individuals with different levels of education. Future studies should aim for a more varied sample to better represent Saudi society. This could include people of different ages (especially those over 25 years of age), various education levels, diverse work backgrounds, and participants from multiple cities and rural areas.

In conclusion, our findings suggest that UPF consumption is associated with excess weight, obesity, and abdominal obesity among Saudi adults. Higher UPF consumption was associated with higher odds of having a BMI of ≥25 kg/m^2^ and abdominal obesity. Both men and women in the highest UPF intake group had significantly higher anthropometric measurements compared to those in the lowest intake group. In terms of daily nutrient intake, higher energy intake from UPF was associated with poor dietary quality, which was characterized by a higher intake of total fat and free sugar and lower intake of MD types. This is the first study to explore this association in Saudi Arabia, validating findings from Brazil, France, Spain, the United States, the United Kingdom, Korea, and China. Additionally, further research is needed to understand better the biological, social, and cultural determinants of potential sex disparities. Future studies should explore populations worldwide, addressing context-specific magnitudes and drivers of UPF consumption and obesity. Mechanistic research is essential to clarify the causal pathways underlying the relationship between food processing and obesity. In considering UPF over-consumption as an important risk factor for non-communicable diseases, overweight, and obesity, our results reinforce the importance of public-health strategies to improve the population’s health by promoting MD as a sustainable diet and limiting the intake of UPF, which is also proposed by the WHO. Our findings suggest that UPF consumption may affect diet quality, because each increase in UPF consumption negatively impacts the nutrient intake among Saudi adults. This evidence can guide policymakers in developing dietary recommendations at the community and clinical levels. More longitudinal studies and randomized controlled trials are necessary to identify the mechanisms linking UPF consumption and obesity. The need for additional research in this field is evident and offers an exciting opportunity for researchers and professionals.

## Data Availability

The original contributions presented in the study are included in the article/Supplementary material, further inquiries can be directed to the corresponding author.
