# Supplementary material for: Ultra-processed food intake and its association with obesity risk factors, Mediterranean diet, and nutrient intake of adults
Source: Front Nutr. 2025 Jul 24;12:1577431. doi: 10.3389/fnut.2025.1577431 (PMC12328165; doi:10.3389/fnut.2025.1577431)
Supplement: Supplementary file 1 [file Table_1.docx]

# Supplementary Material

**Table S1.** NOVA food groups: definition according to the extent and purpose of food processing, with examples. (Monteiro et al., 2019)

| NOVA group | Definition | Examples |
| --- | --- | --- |
| 1) Unprocessed or minimally processed foods | Unprocessed: edible parts of plants (fruits, seeds, leaves, stems, roots, tubers) or of animals (muscle, offal’s, eggs, milk), and fungi, algae and water, after separation from nature.  Minimally processed: unprocessed foods altered by industrial processes such as removal of inedible or unwanted parts, drying, crushing, grinding, fractioning, roasting, boiling, pasteurisation, refrigeration, freezing, placing in containers, vacuum packaging, non-alcoholic fermentation, and other methods that do not add salt, sugar, oils or fats or other food substances to the original food. The main aim of these processes is to extend the life of unprocessed foods, enabling their storage for longer use, and, often, to make their preparation easier or more diverse. Infrequently, minimally processed foods contain additives that prolong product duration, protect original properties or prevent proliferation of microorganisms. | Fresh, squeezed, chilled, frozen, or dried fruits and leafy and root vegetables; grains such as brown, parboiled or white rice, corn cob or kernel, wheat berry or grain; legumes such as beans, lentils, and chickpeas; starchy roots and tubers such as potatoes, sweet potatoes and cassava; fungi such as fresh or dried mushrooms; meat, poultry, fish and seafood, whole or in the form of steaks, fillets and other cuts, fresh or chilled or frozen; eggs; fresh or pasteurized milk; fresh or pasteurised fruit or vegetable juices (with no added sugar, sweeteners or flavours); grits, flakes or flour made from corn, wheat, oats, or cassava; tree and ground nuts and other oily seeds (with no added salt or sugar); herbs and spices used in culinary preparations, such as thyme, oregano, mint, pepper, cloves and cinnamon, whole or powdered, fresh or dried; fresh or pasteurized plain yoghurt; tea, coffee, and drinking water. Also includes foods made up from two or more items in this group, such as dried mixed fruits, granola made from cereals, nuts and dried fruits with no added sugar, honey or oil; pasta, couscous and polenta made with flours, flakes or grits and water; and foods with vitamins and minerals added generally to replace nutrients lost during processing, such as wheat or corn flour fortified with iron and folic acid. |
| 2) Processed culinary ingredients | Substances obtained directly from group 1 foods or from nature by industrial processes such as pressing, centrifuging, refining, extracting or mining. Their use is in the preparation, seasoning and cooking of group 1 foods. These products may contain additives that prolong product duration, protect original properties or prevent proliferation of microorganisms. | Vegetable oils crushed from seeds, nuts or fruits (notably olives); butter and lard obtained from milk and pork; sugar and molasses obtained from cane or beet; honey extracted from combs and syrup from maple trees; starches extracted from corn and other plants, and salt mined or from seawater, vegetable oils with added antioxidants, and table salt with added drying agents. Includes products consisting of two group 2 items, such as salted butter, and group 2 items with added vitamins or minerals, such as iodised salt. |
| 3) Processed foods | Products made by adding salt, oil, sugar or other group 2 ingredients to group 1 foods, using preservation methods such as canning and bottling, and, in the case of breads and cheeses, using non-alcoholic fermentation. Processes and ingredients here aim to increase the durability of group 1 foods and make them more enjoyable by modifying or enhancing their sensory qualities. These products may contain additives that prolong product duration, protect original properties or prevent proliferation of microorganisms. | Canned or bottled vegetables and legumes in brine; salted or sugared nuts and seeds; salted, dried, cured, or smoked meats and fish; canned fish (with or without added preservatives); fruits in syrup (with or without added antioxidants); freshly made unpackaged breads and cheeses. |
| 4) Ultra-processed foods | Formulations of ingredients, mostly of exclusive industrial use, that result from a series of industrial processes (hence ‘ultra-processed’), many requiring sophisticated equipment and technology. Processes enabling the manufacture of ultra-processed foods include the fractioning of whole foods into substances, chemical modifications of these substances, assembly of unmodified and modified food substances using industrial techniques such as extrusion, moulding and pre-frying, frequent application of additives whose function is to make the final product palatable or hyper-palatable (‘cosmetic additives’), and sophisticated packaging, usually with synthetic materials. Ingredients often include sugar, oils and fats, and salt, generally in combination; substances that are sources of energy and nutrients but of no or rare culinary use such as high fructose corn syrup, hydrogenated or interesterified oils, and protein isolates; cosmetic additives such as flavours, flavour enhancers, colours, emulsifiers, sweeteners, thickeners, and anti-foaming, bulking, carbonating, foaming, gelling, and glazing agents; and additives that prolong product duration, protect original properties or prevent proliferation of microorganisms. Processes and ingredients used to manufacture ultra-processed foods are designed to create highly profitable products (low-cost ingredients, long shelf-life, emphatic branding), convenient (ready-to-consume) hyper-palatable snacked products liable to displace all other NOVA food groups, notably group 1 foods. | Carbonated soft drinks; sweet or savoury packaged snacks; chocolate, candies (confectionery); ice-cream; mass-produced packaged breads and buns; margarines and other spreads; cookies (biscuits), pastries, cakes, and cake mixes; breakfast ‘cereals’, ‘cereal’ and ‘energy’ bars; ‘energy’ drinks; milk drinks, ‘fruit’ yoghurts and ‘fruit’ drinks; ‘cocoa’ drinks; ‘instant’ sauces; infant formulas, follow-on milks, other baby products; ‘health’ and ‘slimming’ products such as meal replacement shakes and powders. Many ready to heat products including pre-prepared pies and pasta and pizza dishes; poultry and fish ‘nuggets’ and ‘sticks’, sausages, burgers, hot dogs, and other reconstituted meat products, and powdered and packaged ‘instant’ soups, noodles and desserts. |
